# SARS-CoV-2 S1 Subunit Booster Vaccination Elicits Robust Humoral Immune Responses in Aged Mice

**DOI:** 10.1128/spectrum.04363-22

**Published:** 2023-05-10

**Authors:** Eun Kim, Muhammad S. Khan, Alessandro Ferrari, Shaohua Huang, Josè C. Sammartino, Elena Percivalle, Thomas W. Kenniston, Irene Cassaniti, Fausto Baldanti, Andrea Gambotto

**Affiliations:** a Department of Surgery, University of Pittsburgh School of Medicine, Pittsburgh, Pennsylvania, USA; b Department of Infectious Diseases and Microbiology, University of Pittsburgh Graduate School of Public Health, Pittsburgh, Pennsylvania, USA; c Molecular Virology Unit, Microbiology and Virology Department, IRCCS Policlinico San Matteo, Pavia, Italy; d Department of Clinical, Surgical, Diagnostic and Pediatric Sciences, University of Pavia, Pavia, Italy; e UPMC Hillman Cancer Center, Pittsburgh, Pennsylvania, USA; f Department of Medicine, Division of Infectious Disease, University of Pittsburgh School of Medicine, Pittsburgh, Pennsylvania, USA; g Department of Microbiology and Molecular Genetics, University of Pittsburgh School of Medicine, Pittsburgh, Pennsylvania, USA; Institute of Microbiology, Chinese Academy of Sciences

**Keywords:** COVID-19, SARS-CoV-2, S1 recombinant protein, adenovirus-vectored vaccine, subunit vaccine, prime-boost, adenovirus-based vaccine, S1 protein subunit vaccine

## Abstract

The emergence of severe acute respiratory syndrome coronavirus 2 (SARS-CoV-2) variants has raised concerns about reduced vaccine effectiveness and the increased risk of infection, and while repeated homologous booster shots are recommended for elderly and immunocompromised individuals, they cannot completely protect against breakthrough infections. In our previous study, we assessed the immunogenicity of an adenovirus-based vaccine expressing SARS-CoV-2 S1 (Ad5.S1) in mice, which induced robust humoral and cellular immune responses (E. Kim, F. J. Weisel, S. C. Balmert, M. S. Khan, et al., Eur J Immunol 51:1774–1784, 2021, https://doi.org/10.1002/eji.202149167). In this follow-up study, we found that the mice had high titers of anti-S1 antibodies 1 year after vaccination, and one booster dose of the nonadjuvanted rS1Beta (recombinant S1 protein of SARS-CoV-2 Beta [B.1.351]) subunit vaccine was effective at stimulating strong long-lived S1-specific immune responses and inducing significantly high neutralizing antibodies against Wuhan, Beta, and Delta strains, with 3.6- to 19.5-fold increases. Importantly, the booster dose also elicited cross-reactive antibodies, resulting in angiotensin-converting enzyme 2 (ACE2) binding inhibition against spikes of SARS-CoV-2, including Omicron variants, persisting for >28 weeks after booster vaccination. Interestingly, the levels of neutralizing antibodies were correlated not only with the level of S1 binding IgG but also with ACE2 inhibition. Our findings suggest that the rS1Beta subunit vaccine candidate as a booster has the potential to offer cross-neutralization against broad variants and has important implications for the vaccine control of newly emerging breakthrough SARS-CoV-2 variants in elderly individuals primed with adenovirus-based vaccines like AZD1222 and Ad26.COV2.S.

**IMPORTANCE** Vaccines have significantly reduced the incidences of severe coronavirus disease 2019 (COVID-19) cases and deaths. However, the emergence of SARS-CoV-2 variants has raised concerns about their increased transmissibility and ability to evade neutralizing antibodies, especially among elderly individuals who are at higher risks of mortality and reductions of vaccine effectiveness. To address this, a heterologous booster vaccination strategy has been considered as a solution to protect the elderly population against breakthrough infections caused by emerging variants. This study evaluated the booster effect of an S1 subunit vaccine in aged mice that had been previously primed with adenoviral vaccines, providing valuable preclinical evidence for elderly people vaccinated with the currently approved COVID-19 vaccines. This study confirms the potential for using the S1 subunit vaccine as a booster to enhance cross-neutralizing antibodies against emerging variants of concern.

## INTRODUCTION

Severe acute respiratory syndrome coronavirus 2 (SARS-CoV-2) was identified as the causative agent of coronavirus disease 2019 (COVID-19) in December 2019, leading to the COVID-19 pandemic. The COVID-19 pandemic has resulted in 761 million confirmed cases, 6.8 million reported deaths, and the administration of 13.2 billion vaccine doses worldwide (until 21 March 2023) (1). Six vaccines targeting the spike (S) protein of SARS-CoV-2 (BNT162b2, AZD1222, Ad26.COV2.S, mRNA-1273, NVX-CoV2373, and Ad5-nCoV) have been approved by the World Health Organization (WHO), greatly reducing the rates of severe disease and death (2). However, the evolution of SARS-CoV-2 has given rise to multiple variants, including SARS-CoV-2 variants of concern (VOCs) such as Alpha (B.1.1.7), Beta (B.1.351), Gamma (P.1), Delta (B.1.617.2), Omicron (B.1.1.529), and the most recent Omicron subvariant (XBB.1.5). These variants are characterized by their potential for increased transmissibility, their ability to escape neutralizing antibodies, and the reduced effectiveness of vaccinations or antibody treatments (3).

It is clear that age is the most significant risk factor for death due to COVID-19 (4–6). Recent reports suggested that individuals over 65 years old account for 80% of COVID-19 hospitalizations and have a 20-fold-higher COVID-19 fatality rate than those under 65 years old (7–9). Among elderly individuals, those aged 80 years or older are at the highest risk of severe COVID-19 (10). Furthermore, elderly individuals have been found to have poor neutralization, which may be due to lower levels of serum IgG, lower levels of somatic hypermutation in B cell selection, and lower levels of interleukin-2 (IL-2)-producing CD4^+^ T cell help than in younger individuals. All of these factors can be overcome by booster vaccination (11). These findings are consistent with previous studies showing a lower immune response in aged mice vaccinated with ChAdOx1 nCov-19 than in younger mice, which was improved by booster dosing (12).

The entry of coronaviruses into host cells is mediated by the interaction between the receptor binding domain (RBD) of the viral S protein and the host receptor angiotensin-converting enzyme 2 (ACE2) through the upper and lower respiratory tracts (13, 14). Neutralizing antibodies against SARS-CoV-2 are effective at blocking this interaction to prevent infection (15, 16). The results of competitive immunoassays for quantifying the inhibition of the spike-ACE2 interaction show high levels of concordance with the results of neutralizing tests (17, 18). VOCs have mutations or deletions in the spike protein, with some mutations occurring in the RBD, resulting in the highest level of resistance to vaccine-induced and infection-acquired immunity. In response to the rapid evolution of SARS-CoV-2 and the global circulation of VOCs, booster injections have been considered to protect against breakthrough infections with new emerging variants. Evaluations of booster immunization have been performed in mice, nonhuman primates, and humans (12, 19–22). The findings suggested that the level of neutralizing antibodies is correlated with vaccine efficacy for both mRNA and adenovirus (Ad)-vectored vaccines, and there is likely potential efficacy after boosting (23–26). Of note, ChAdOx1-mRNA vaccination was safe and provided enhanced immunogenicity compared to ChAdOx1-ChAdOx1 vaccination, highlighting that heterologous prime-boost regimens may offer immunological advantages for eliciting strong and long-lasting protection acquired with currently available adenovirus-based vaccines (27–29). Overall, heterologous booster administration has been considered as a solution to protect elderly people from breakthrough infections with new emerging variants.

In our previous study, we assessed the immunogenicity of an adenovirus-based vaccine expressing SARS-CoV-2 S1 (Ad5.S1) in mice. We found that a single immunization with Ad5.S1, via subcutaneous (s.c.) injection or intranasal (i.n.) delivery, induced robust humoral and cellular immune responses (30). Here, we conducted a follow-up study to assess the long-term persistence of immunogenicity and the booster effect of a subunit vaccine in aged mice. For the subunit vaccine, the recombinant S1 protein of SARS-CoV-2 Beta (B.1.351) (rS1Beta) was selected because it showed the highest number of breakthrough infections against the Wuhan (WU)-based vaccines (31, 32) before the COVID-19 waves caused by Omicron variants, which lately have shown even higher levels of vaccine escape. In the present study, we evaluated whether mice vaccinated with Ad5.S1 had high titers of anti-S1 antibodies 1 year after immunization compared to phosphate-buffered saline (PBS)-immunized mice. A booster with the rS1Beta subunit vaccine was effective at stimulating strong, long-lived, S1-specific immune responses and inducing significantly high levels of cross-neutralizing antibodies against SARS-CoV-2 variants.

## RESULTS

### Construction and expression of recombinant proteins.

**FIG 1:**
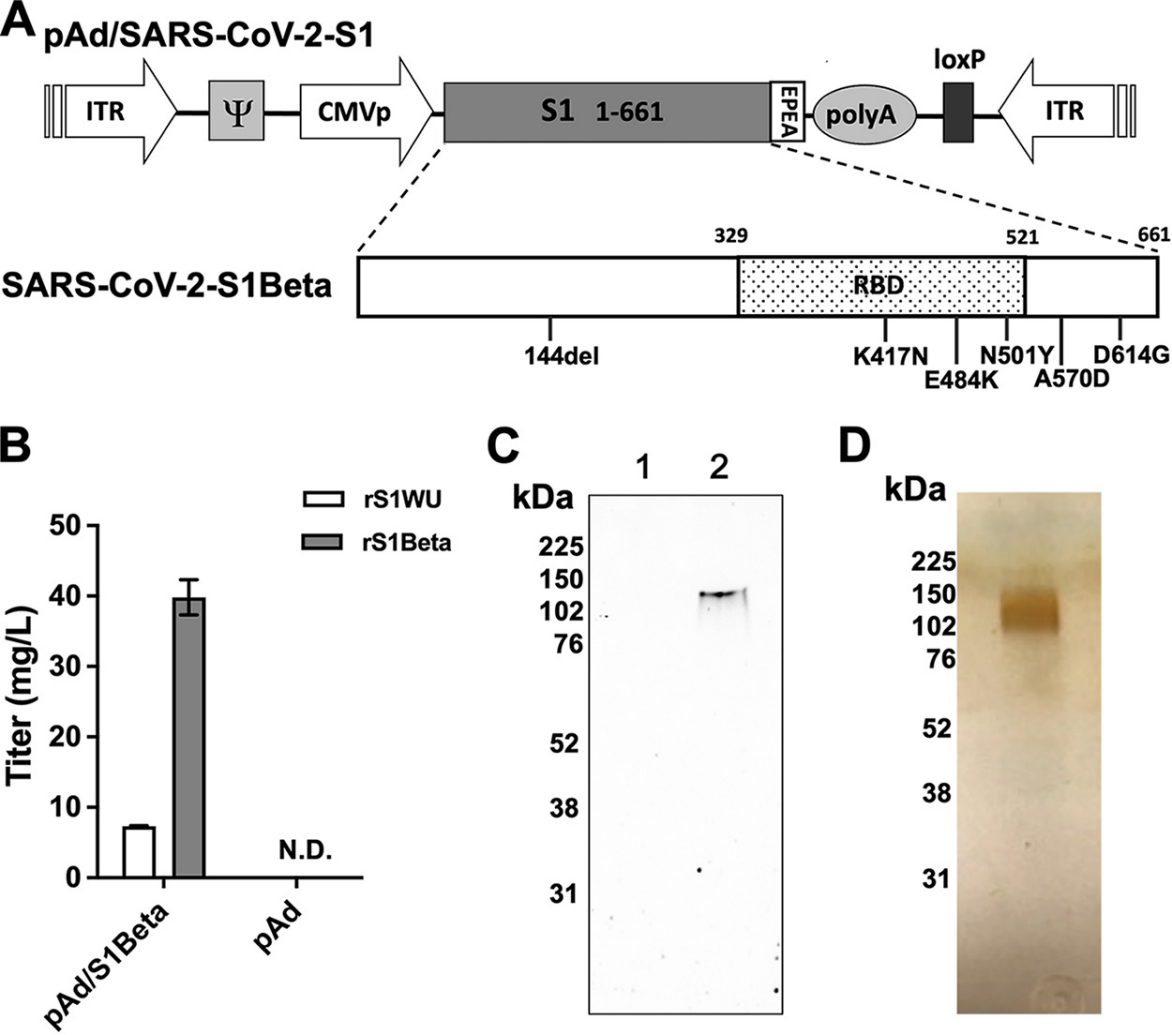
Construction of a recombinant SARS-CoV-2 S1Beta protein-expressing plasmid. (A) Diagram showing a shuttle vector carrying the codon-optimized SARS-CoV-2 S1 gene of Beta (1.351.1) variants encoding N-terminal amino acids 1 to 661 with a C-tag (EPEA, glutamic acid-proline-glutamic acid-alanine). The amino acid changes in the SARS-CoV-2 S1 region from this study are also shown. ITR, inverted terminal repeat; CMVp, cytomegalovirus promoter; RBD, receptor binding domain. (B) The titer of recombinant SARS-CoV-2 S1 proteins was determined by a sandwich ELISA with the supernatant of Expi293 cells transfected with pAd/SARS-CoV-2-S1Beta (pAd/S1Beta) based on the standard of rS1Wuhan (WU) or rS1Beta. N.D., not determined. (C) Detection of the SARS-CoV-2 S1 proteins by Western blotting with the supernatant of Expi293 cells transfected with pAd/S1Beta using a rabbit anti-SARS-CoV-2 spike polyclonal antibody (lane 2). As a negative control, mock-transfected cells were treated the same (lane 1). The supernatants were resolved on an SDS–10% polyacrylamide gel after being boiled in 2% SDS sample buffer with β-mercaptoethanol (β-ME). (D) Purified Expi293 cell-derived rS1Beta (300 ng) was analyzed using a silver-stained reducing SDS-PAGE gel.

To produce recombinant proteins of SARS-CoV-2 S1, pAd/S1Beta was generated by subcloning the codon-optimized SARS-CoV-2 S1Beta gene with a C-tag into the shuttle vector pAd (GenBank accession number U62024) at the SalI and NotI sites (Fig. 1A). To determine whether rS1Beta proteins were expressed from the plasmid, Expi293 cells were transfected with pAd/S1Beta or pAd as a control. Five days after transfection, the supernatants of Expi293 cells were characterized by a sandwich enzyme-linked immunosorbent assay (ELISA) using a monoclonal antibody (MAb) pair against SARS-CoV-2 Wuhan (WU) (Fig. 1B) and Western blot analysis (Fig. 1C). As shown in Fig. 1B, the titers of recombinant rS1Beta proteins expressed in Expi293 cells were approximately 7.3 mg/L based on the standard of rS1WU and about 40.0 mg/L based on the standard of rS1Beta, while the rS1Beta protein was not detected in Expi293 cells transfected with the control vector pAd. The rS1Beta protein was separated by 10% sodium dodecyl sulfate-polyacrylamide gel electrophoresis (SDS-PAGE) and was recognized by a polyclonal anti-SARS-CoV-2 spike antibody at the expected glycosylated monomeric molecular weight of approximately 110 kDa under denaturing-reducing conditions, while no expression was detected in mock-transfected cells (Fig. 1C). The purified rS1Beta protein, using a C-tagXL affinity matrix, was detected by silver staining (Fig. 1D).

### Rapid recall of S1-specific binding antibodies after a booster.

**FIG 2:**
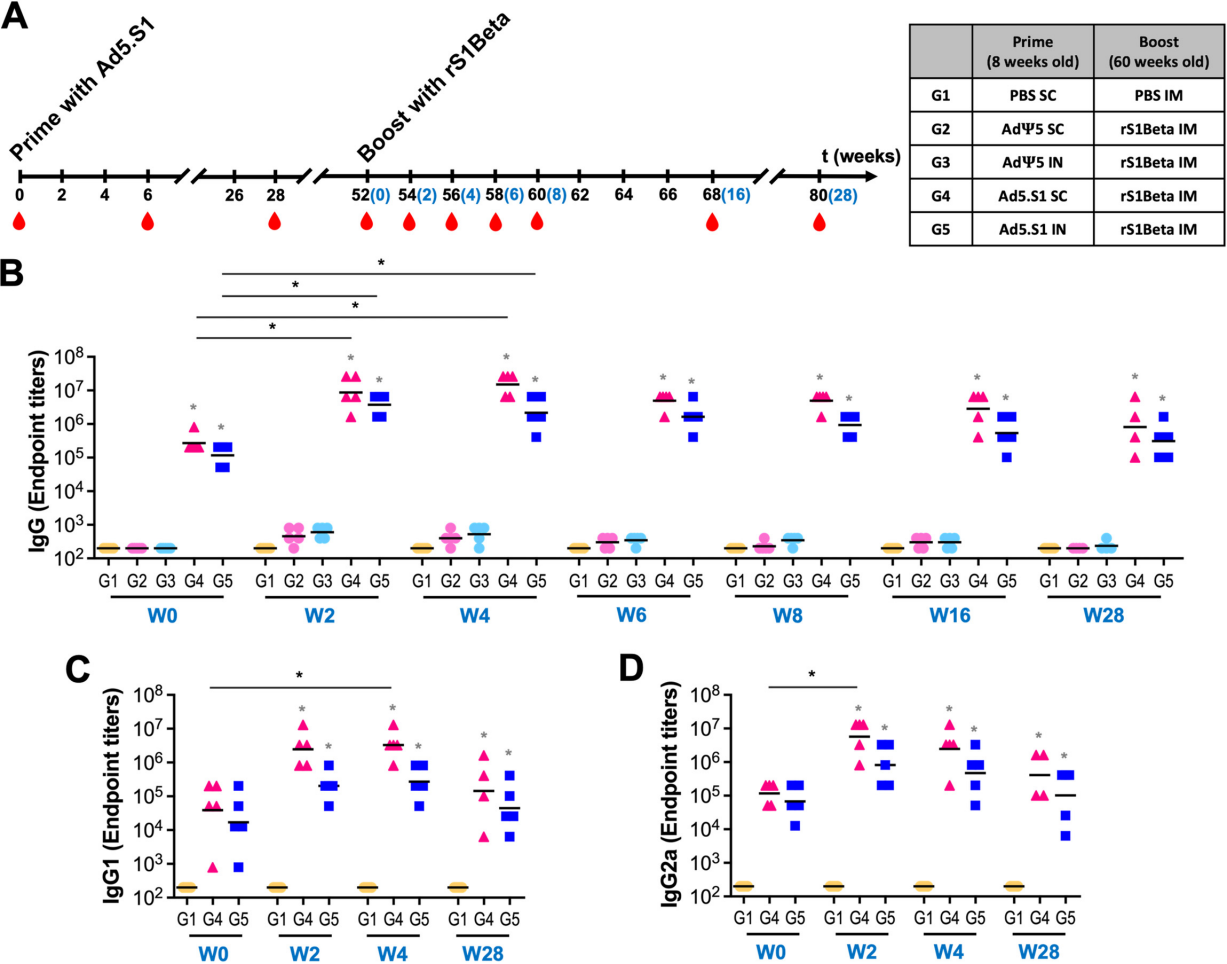
Prime-boost immunization with SARS-CoV-2 adenoviral vaccine-subunit proteins in BALB/c mice. (A) Schedule of immunization and blood sampling for IgG endpoint titration and explanation of animal groups. BALB/c mice were primed with 1.5 × 10^10^ viral particles (vp) of an adenoviral vaccine (Ad5.S1 or AdΨ5) s.c. or i.n. and PBS as a negative control at 8 weeks of age and boosted with 15 μg of SARS-CoV-2 S1Beta recombinant proteins i.m. at a 1-year interval (60 weeks old). The black and blue numbers represent weeks after prime and boost immunizations, respectively. The immune responses were assessed at weeks 0, 2, 4, 6, 8, 16, and 28 postboost (*n* = 5 per group, except for G4 at week 80 [*n* = 4]). (B to D) Reciprocal serum endpoint dilutions of SARS-CoV-2 S1-specific antibodies were measured by an ELISA to determine IgG (at week 0 [W0] and weeks 2, 4, 6, 8, 16, and 28 postboost) from G1 (peach circles), G2 (light-pink circles), G3 (light-blue circles), G4 (pink triangles), and G5 (blue squares) (B) and IgG1 (C) and IgG2a (D) (at weeks 0, 2, 4, and 28 postboost) from G1, G4, and G5. Horizontal lines represent geometric mean antibody endpoint titers (GMTs). Significance was determined by a Kruskal-Wallis test followed by Dunn’s multiple-comparison test (*, *P < *0.05). Gray asterisks represent statistical differences compared with G1 (PBS group).

In our previous study, we evaluated the immunogenicity of the adenoviral vaccine until week 24 (30). To assess the long-term persistence of immunogenicity, we first determined the antigen-specific IgG antibody endpoint titers in the sera of vaccinated mice (groups immunized with Ad5.S1 via either i.n. delivery or s.c. injection) and control mice (PBS-treated group or AdΨ5-immunized groups) at week 52, 1 year after prime vaccination (Fig. 2A). As shown in Fig. 2B, significantly high titers of anti-S1 IgG antibodies were present in the Ad5.S1-vaccinated mouse groups (group 4 [G4], *P* = 0.0016; G5, *P* = 0.0365), even 1 year after vaccination, compared to the AdΨ5-vaccinated mouse groups (G2 and G3) or the PBS group (G1). To assess the booster effect of the subunit vaccine, we collected serum samples from all mice before booster immunization (week 52), immunized animals with 15 μg of rS1Beta intramuscularly (i.m.) at week 52 (60 weeks old) postprime, and collected sera at subsequent weeks until week 28 postboost (Fig. 2A). The endpoint titers of IgG against spike protein S1 subunit (anti-S1) binding antibodies were examined by an ELISA (Fig. 2B). Significantly higher levels of binding antibodies were detected in the Ad5.S1-vaccinated mouse groups (G4 and G5) than in the AdΨ5-vaccinated mouse groups (G2 and G3) or the PBS group (G1) until week 28 (*P < *0.05) after booster vaccination. The changes in the geometric mean titers (GMTs) of the IgG endpoint titers in G4 and G5 compared to those at week 0 were the same at 32-fold at week 2 postboost and diverged to 55.7-fold and 18.4-fold at week 4 postboost, respectively (see Fig. S1A in the supplemental material). Interestingly, the peak IgG endpoint titer appeared at week 4 postboost in G4, while it appeared at week 2 postboost in G5. These recalls were fast after booster vaccination with the rS1Beta subunit vaccine compared with the IgG endpoint titer after prime vaccination (week 6 postprime versus week 2 or 4 postboost) (30). Furthermore, the IgG antibody responses elicited after booster vaccination lasted longer, through week 28 postboost (maximum length of the study to date), than after prime vaccination, as shown by the comparison with the IgG endpoint titers at week 28 postprime or postboost (week 80) (Fig. S1B). The GMT of the IgG endpoint titers of the mouse group primed s.c. (G4) was high compared to that of the mouse group primed i.n. (G5).

Serum samples collected at weeks 0, 2, 4, and 28 postboost were serially diluted to determine SARS-CoV-2 S1-specific IgG1 and IgG2a endpoint titers for each immunization group, indicating a Th2- or a Th1-like response, respectively, using an ELISA (Fig. 2C and D). The levels of induction of S1-specific IgG1 and IgG2a antibodies were significant and similar in G4 and G5 after a booster shot, indicating a balanced Th1/Th2 response. Although there were no significant differences in the S1-specific IgG1 and IgG2a responses at week 0 compared to those of G1, significantly different IgG1 and IgG2a responses were observed for G4 (*P < *0.001 at weeks 2 and 4; *P < *0.05 at week 28 postboost) than for G5 (*P < *0.05 at weeks 2, 4, and 28 postboost) compared with G1. Interestingly, IgG2a (Th1) responses were recalled faster than IgG1 (Th2) responses in both G4 and G5 (peak at week 2 versus week 4 postboost, respectively). These results suggest that booster immunization with the rS1Beta subunit vaccine induced significantly increased S1-specific IgG, IgG1, and IgG2a endpoint titers, which were recalled quickly (Fig. 2B to D) (*P < *0.05 by a Kruskal-Wallis test followed by Dunn’s multiple-comparison test). Furthermore, the elicited IgG, IgG1, and IgG2a antibody responses remained significantly high with respect to the control groups through week 28 postboost (maximum length of the study to date) compared to postprime (Fig. 2 and Fig. S1). Together, these results suggest that a booster could generate robust, balanced, and long-lived S1-specific antibody responses in aged mice primed with Ad5.S1 via either s.c. delivery or i.n. administration 1 year previously.

### Neutralizing antibody levels after a booster.

**FIG 3:**
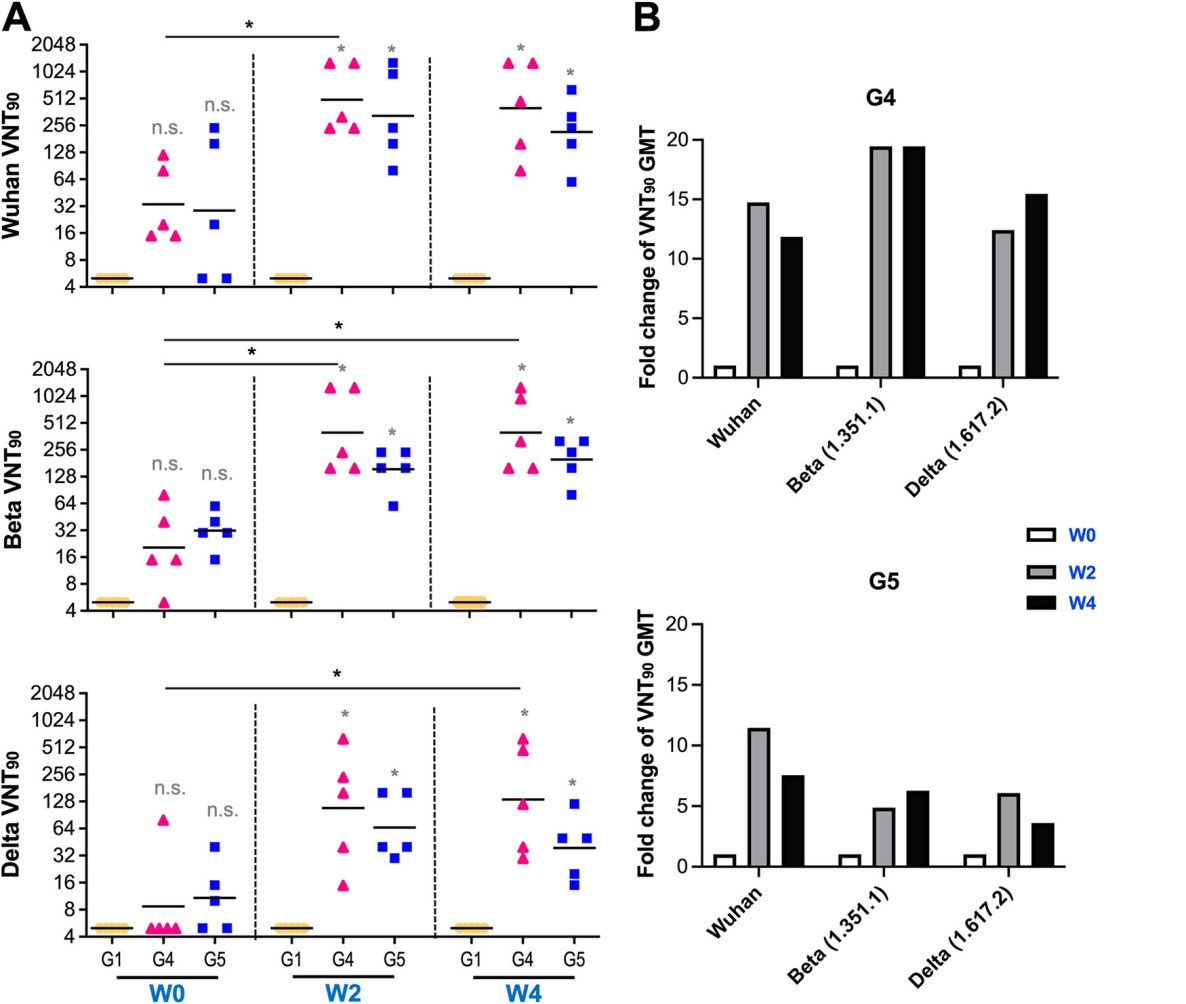
Neutralizing antibody responses in mice after a boost. BALB/c mice (*n* = 5 mice per group) were prime immunized s.c. or i.n. with 1.5 × 10^10^ vp of Ad5.SARS-CoV-2-S1 (Ad5.S1), with PBS as a negative control, at 8 weeks of age and boosted with 15 μg of SARS-CoV-2 S1Beta recombinant proteins i.m. at week 52 postprime (60 weeks old). (A) Neutralizing antibody titers from G1 (peach circles), G4 (pink triangles), and G5 (blue squares) against SARS-CoV-2 Wuhan, Beta (B.1.351), and Delta (B.1.617.2) variants were measured using a VNT_90_ assay at weeks 0, 2, and 4 postboost. Serum titers resulting in a 90% reduction in cytopathic effect compared to the virus control are reported. Horizontal lines represent geometric mean neutralizing antibody titers. Groups were compared by a Kruskal-Wallis test at each time point, followed by Dunn’s multiple-comparison test. Significant differences are indicated by asterisks (*, *P* < 0.05; n.s., not significant). The minimal titer tested was 10, and undetectable titers (those with NT_90_ values of <10) were assigned a value of 5. Gray asterisks represent statistical differences compared with the PBS group (G1). (B) Fold changes in the GMTs of the VNT_90_ values against Wuhan, Beta (B.1.351), and Delta (B.1.617.2) in G4 and G5 after a booster (weeks 2 and 4), relative to those with a prebooster (week 0).

To evaluate the presence of long-term and booster-generated SARS-CoV-2-specific neutralizing antibodies, we used a microneutralization assay (90% virus neutralization titer [VNT_90_]) to test the ability of sera from immunized mice to neutralize the infectivity of SARS-CoV-2 variants, including Wuhan, Beta (B.1.351), and Delta (B.1.617.2) variants, as shown in Fig. 3A. SARS-CoV-2-neutralizating antibodies were detected in the Ad5.S1-vaccinated groups (G4 and G5), even 1 year after prime vaccination, with no significant differences compared to the PBS group (G1). The GMTs of the VNT_90_ in G4 and G5 were 33.7 and 28.6 against Wuhan, 20.5 and 31.8 against Beta (B.1.351), and 8.7 and 10.8 against Delta at week 0 (week 52 postprime), respectively. These results clearly showed low levels of neutralization of the Delta (B.1.617.2) variant compared to the other variants.

After booster vaccination, the SARS-CoV-2-neutralizing activities at week 2 and week 4 were statistically significant (Fig. 3A) (*P* < 0.05 by a Kruskal-Wallis test followed by Dunn’s multiple-comparison test) compared to the control groups, with no significant differences between each other. The fold changes in the GMTs of the VNT_90_s against Wuhan, Beta (B.1.351), and Delta (B.1.617.2) in G4 compared to those at week 0 were 14.7-, 19.5-, and 12.4-fold at week 2 and 11.8-, 19.5-, and 15.5-fold at week 4, respectively (Fig. 3B). Those for G5 were 11.5-, 4.9-, and 6.1-fold at week 2 and 7.6-, 6.3-, and 3.6-fold at week 4, respectively. These fold changes in the GMTs of the VNT_90_s were statistically significant in G4 against all variants, with no significant differences compared to G5. Interestingly, the highest fold change was against Beta (B.1.351) in G4, while it was against Wuhan in G5. There were no detected neutralizing antibody responses in the sera of mice in the AdΨ5-vaccinated groups (G2 and G3) after the booster (data not shown).

To assess the correlations between the levels of S1 binding IgG endpoint titers and neutralizing antibodies, we performed correlation analyses on log-transformed data. We found a positive correlation between S1 binding IgG titers and VNT_90_ values in all animals from G1, G4, and G5 at weeks 0, 2, and 4 postboost (Spearman’s correlation coefficient [*r*] = 0.9177 [95% confidence interval {CI}, 0.8462 to 0.9567] for Wuhan, *r *= 0.9498 [95% CI, 0.9047 to 0.9738] for Beta, and *r *= 0.8875 [95% CI, 0.7925 to 0.9404] for Delta) (Fig. S2). The highest to lowest correlations between S1 binding IgG endpoint titers and neutralizing antibodies were for Beta, Wuhan, and Delta, respectively, with Beta being a subunit vaccine booster variant.

### ACE2 binding inhibition.

**FIG 4:**
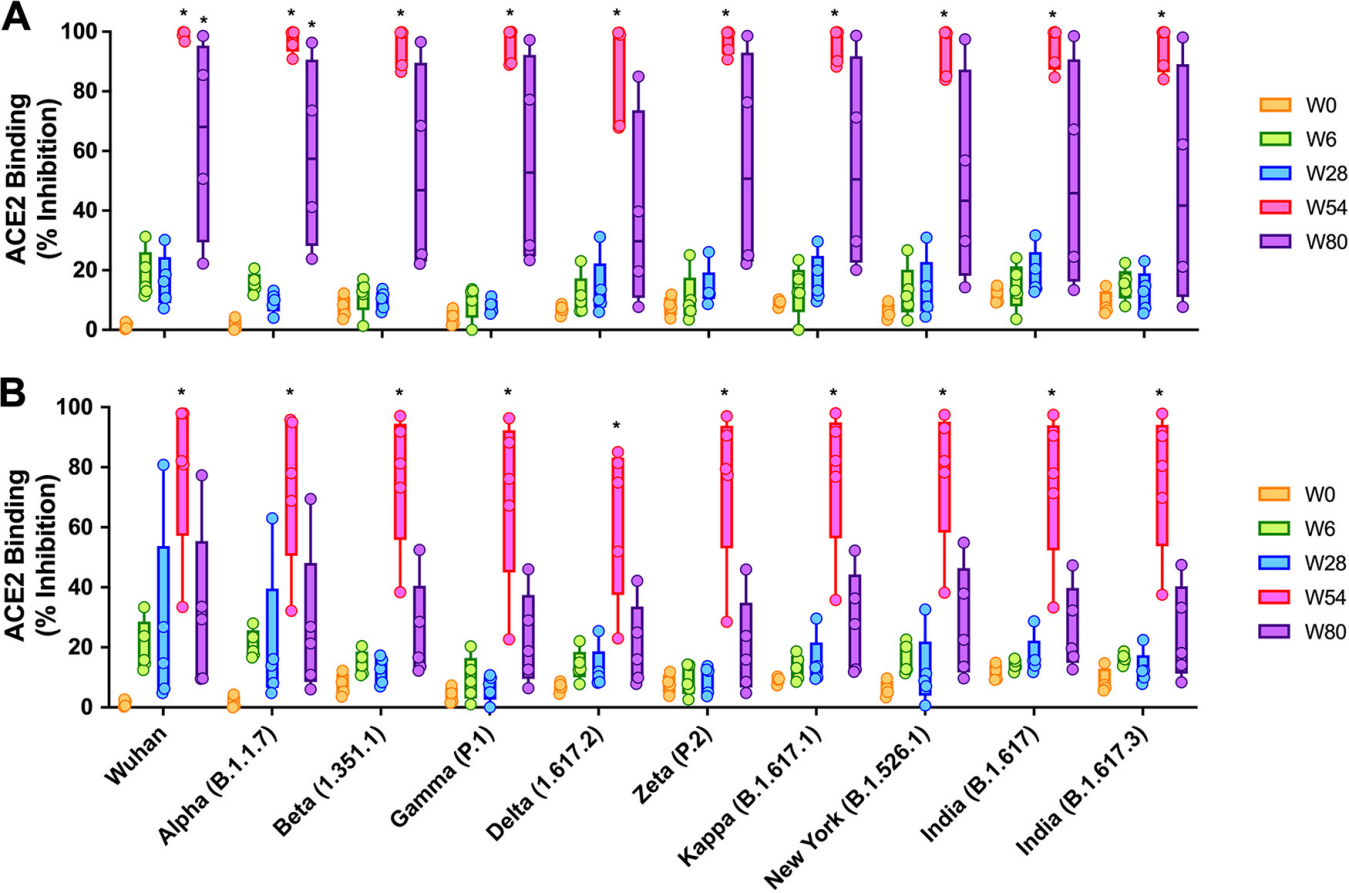
Percent ACE binding inhibition of neutralizing antibodies against SARS-CoV-2 variants. Antibodies in sera capable of neutralizing the interaction between the spike proteins of SARS-CoV-2 Wuhan, Alpha (B.1.1.7), Beta (B.1.351), Gamma (P.1), Delta (B.1.617.2), Zeta (P.2), Kappa (B.1.617.1), New York (B.1.516.1), and India (B.1.617 and B.1.617.3) variants and ACE2 were examined in all animals from G4 (A) and G5 (B) at weeks 0, 6, 28, 54, and 80 postprime. Serum samples were diluted 1:100 before being added to the V-Plex plates. Box-and-whisker plots represent the medians and upper and lower quartiles (boxes) with minima and maxima (whiskers). There is no significant difference among all of the variants at the same time points, either before or after a booster. Asterisks represent statistical differences compared with preimmunized sera.

Additional tests were conducted to evaluate the ability of serum antibodies to inhibit the binding between ACE2 and the trimeric spike proteins of SARS-CoV-2 variants. We used V-plex SARS-CoV-2 (ACE2) kit panel 18, which included Wuhan, Alpha (B.1.1.7), Beta (B.1.351), Gamma (P.1), Delta (B.1.617.2), Zeta (P.2), Kappa (B.1.617.1), New York (B.1.516.1), and India (B.1.617 and B.1.617.3). The antibodies’ abilities to neutralize the interaction between the spike proteins of SARS-CoV-2 variants and ACE2 were examined in all animals from G4 (Fig. 4A) and G5 (Fig. 4B) at weeks 0, 6, 28, 54, and 80 postprime at a dilution of 1:100. The ACE2-inhibitory activities of the sera from G4 against all variants were on average 13.2% ± 6.98%, 13.3% ± 6.83%, 94.9% ± 6.80%, and 52.9% ± 36.47% at weeks 6, 28, 54, and 80, respectively. Those from G5 were on average 14.7% ± 4.82%, 14.7% ± 10.87%, 74.1% ± 25.38%, and 25.2% ± 18.11%, respectively, with 6.4% ± 2.65% at week 0. Overall, the median percent inhibition was lower for all variants than for wild-type Wuhan. Interestingly, the differences for all variants reached statistical significance in both G4 and G5 at week 2 postboost (week 54 postprime) compared to week 0 (Fig. 4A and B). The inhibitions of the Wuhan and Alpha (B.1.1.7) spike proteins by vaccine-induced antibodies at week 80 were significantly different from those at week 0 in G4 only. The increase and decrease in the percent inhibitions of the different variants followed the same trends for both groups. The highest and lowest percent inhibitions of neutralizing antibodies compared to Wuhan were observed for Alpha (B.1.1.7) and Delta (B.1.617.2), respectively.

**FIG 5:**
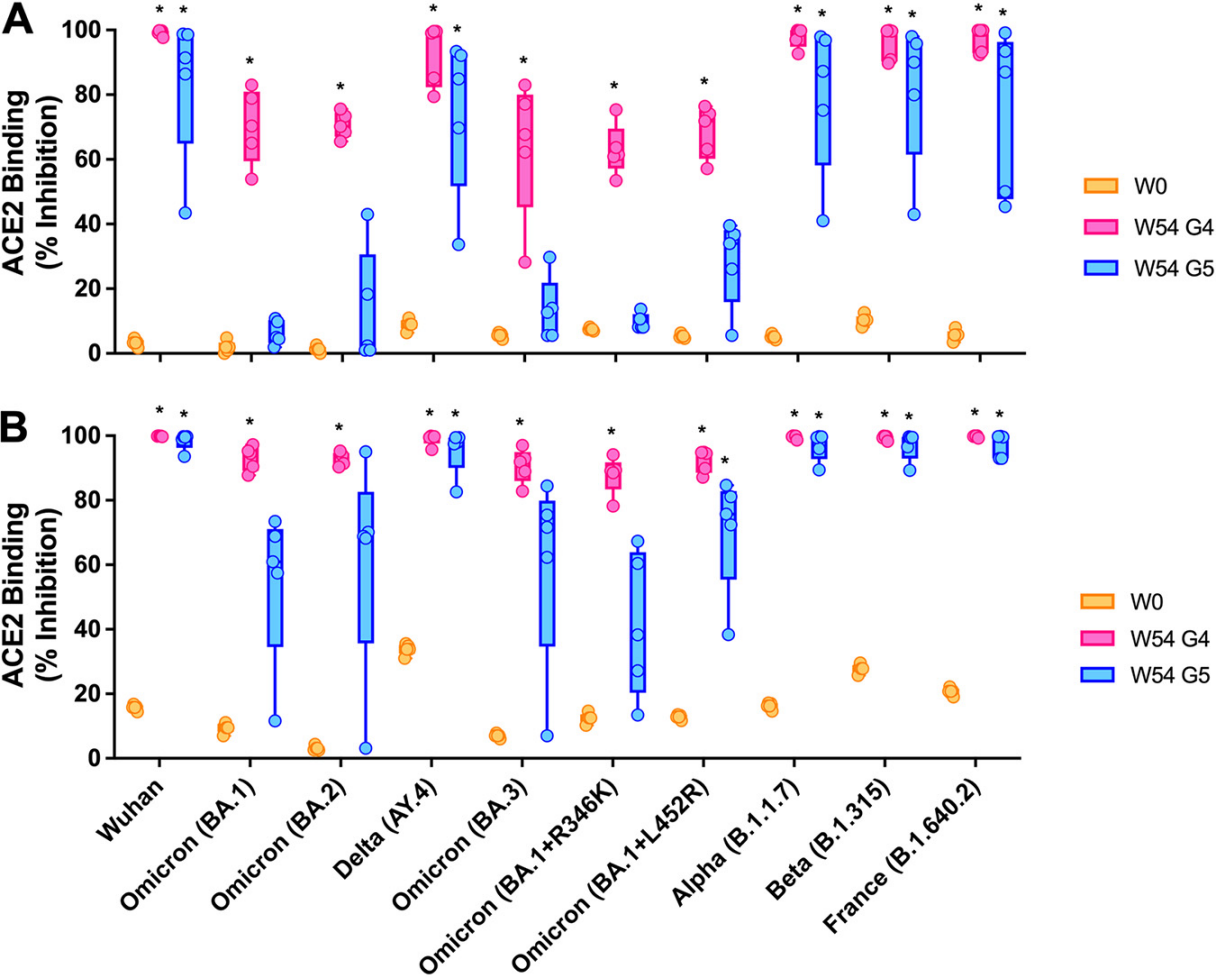
Percent ACE binding inhibition of neutralizing antibodies against SARS-CoV-2 Omicron variants. Antibodies in sera capable of neutralizing the interaction between the spike proteins of SARS-CoV-2 Wuhan, Omicron (BA.1), Omicron subvariants (BA.2, BA.3, BA.1+R346K, and BA.1+L452R), the Delta lineage (AY.4), Alpha (B.1.1.7), Beta (B.1.351), and France (B.1.640.2) and ACE2 were examined at week 0 and week 54 postprime from G4 and G5. Serum samples were diluted 1:100 (A) and 1:25 (B) before being added to the V-Plex plates. Box-and-whisker plots represent the medians and upper and lower quartiles (boxes) with minima and maxima (whiskers). Asterisks represent statistical differences compared with preimmunized sera.

To further assess the neutralizing abilities of antibodies after booster vaccination against Omicron (BA.1) and its subvariants, we used Meso Scale Discovery (MSD) V-Plex SARS-CoV-2 (ACE2) kit panel 25, which includes Wuhan, Omicron (BA.1), Omicron subvariants (BA.2, BA.3, BA.1+R346K, and BA.1+L452R), the Delta lineage (AY.4), Alpha (B.1.1.7), Beta (B.1.351), and France (B.1.640.2) (Fig. 5). The ACE2 binding inhibitions of sera from G4 and G5 were examined at week 54 (week 2 postboost) and compared to that of preimmunized sera at a 1:100 dilution (Fig. 5A). The ACE2 binding inhibitions of antibodies from G4 sera at week 54 were significantly increased compared to those at week 0 for all Omicron variants at a 1:100 dilution, while antibodies from G5 sera showed very low ACE2 binding inhibition. They were significantly increased only for the spikes of Wuhan, Delta, Alpha, Beta, and France compared to those at week 0. To further investigate the boost-induced neutralizing activities of G5 sera against Omicron variants, mouse sera were diluted 1:25 (Fig. 5B). The ACE2 binding inhibition of G5 week 54 sera was significantly increased compared to that of week 0 sera for BA.1+L452R spike at a 1:25 dilution. While not statistically significant compared to week 0, G5 sera demonstrated moderate ACE2 binding inhibition of BA.1, BA.2, BA.3, and BA.1+R346K spikes. The ACE2-inhibitory activities of G5 week 54 sera against BA.1, BA.2, BA.3, BA.1+R346K, and BA.1+L452R variants were on average 6.4% ± 3.38%, 13.1% ± 16.29%, 13.5% ± 8.81%, 9.7% ± 2.18%, and 28.4% ± 12.24% at a 1:100 dilution and 54.5% ± 22.15%, 61.1% ± 30.66%, 60.2% ± 27.51%, 41.3% ± 20.13%, and 70.4% ± 16.58% at a 1:25 dilution, respectively.

### Correlations between the levels of ACE2 inhibition and neutralizing antibodies.

**FIG 6:**
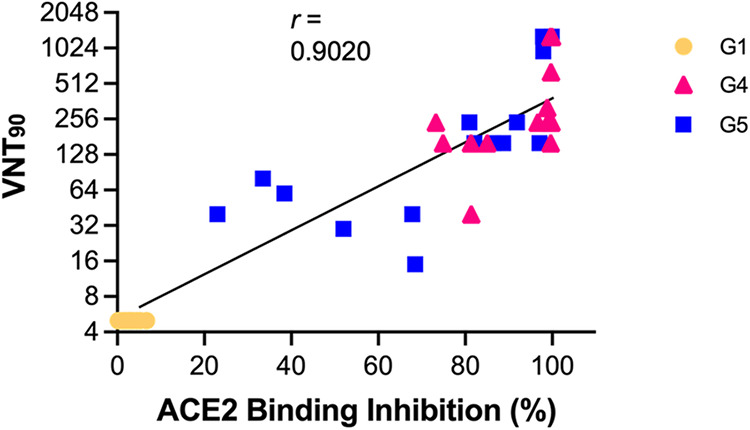
Correlation between VNT_90_ values and ACE2 binding inhibition. The correlations between VNT_90_ (log_2_) values against Wuhan, Beta (B.1.351), and Delta (B.1.617.2) and ACE2 binding inhibition (percent) of 1:100-diluted sera from all animals from G1, G4, and G5 at week 2 postboost are shown. The lines represent the regression lines for all samples. Each symbol represents an individual mouse. Correlation analysis and calculation of Spearman’s correlation coefficients were performed using GraphPad Prism v9.

After the receipt of a booster, ACE2 binding inhibition and VNT_90_ values against Wuhan, Beta (B.1.351), and Delta (B.1.617.2) increased significantly compared to those of the prevaccinated sera, with no differences being found among the variants. To determine the correlations between the levels of ACE2 inhibition and the levels of neutralizing antibodies, we performed correlation analyses on ACE2 inhibition of 1:100-diluted mouse sera and log-transformed VNT_90_ data for Wuhan, Alpha (B.1.1.7), and Delta (B.1.617.2). We found a positive correlation between V-Plex ACE2 inhibition and VNT_90_ values for all animals from G1, G4, and G5 at week 2 postboost (*r *= 0.9025 [95% CI, 0.8190 to 0.9486]; *P* < 0.0001) (Fig. 6). Spearman’s correlation coefficients were lower when the analysis was performed with 1:400-diluted mouse sera (*r *= 0.7802 [95% CI, 0.6132 to 0.8804]; *P* < 0.0001) (Fig. S3). Changes in ACE2 binding inhibition at weeks 6, 28, 54, and 80 postprime against the Wuhan spike protein were dependent on the dilution factor, showing a pattern similar to those for the other variants (Fig. S1C). Taken together, a single dose of the nonadjuvanted recombinant S1 protein subunit vaccine as a booster induced broadly cross-reactive neutralizing antibodies against a wide range of SARS-CoV-2 variants, including Omicron, in aged mice primed with Ad5.S1 s.c., and the neutralizing antibody titer was correlated with the inhibition of spike-ACE2 binding.

## DISCUSSION

We previously reported that a single immunization of BALB/c mice (8 weeks old) with our adenovirus-based COVID-19 vaccine (Ad5.S1) via either i.n. or s.c. delivery elicited robust S1-specific humoral and cellular immune responses in mice. In this study, we demonstrated the long-term persistence of immunogenicity after prime vaccination for up to 1 year. Additionally, we demonstrate that a booster of nonadjuvanted rS1Beta in aged mice (60 weeks old) primed with Ad5.S1 1 year previously induces robust, balanced, and long-lasting IgG antibodies and neutralizing antibodies that broadly cross-react with SARS-CoV-2 variants and correlate with ACE2-spike interaction inhibition.

There were very low levels of antibody responses in the sera from mice in the AdΨ5-vaccinated groups (G2 and G3) after a subunit booster injection at week 52 postprime, which might be explained by the age of the mice at the time of the single immunization (Fig. 2A). Indeed, vaccinated aged mice elicited a low level of immune responses compared to vaccinated young mice, which was found to be due to the low frequencies of IgG- and interferon gamma (IFN-γ)-secreting cells in vaccinated aged mice (33). These results were parallel to previous findings that older individuals have lower levels of immune responses to approved COVID-19 vaccines than younger individuals (9, 11, 33, 34). Especially, lower serum IgG levels against SARS-CoV-2 in elderly people were due to a lower proportion of peripheral spike-specific memory B cells (11).

We have not compared the immune responses between young and aged mice using the same immunogens and delivery routes. However, based on our previous experiment with Ad5.S1N-rS1WU or Ad5.S1N-rS1Beta (intramuscular [i.m.] prime-i.m. boost regimen at a 3-week interval in 6-week-old mice), the GMTs of the IgG endpoint titers at weeks 2 and 4 postboost were increased 9.2-fold and 16.0-fold, respectively, compared to those at week 0 in both groups (35). In the case of the long interval of 1 year in this study, the change in the GMT of the IgG endpoint titers in G4 and G5 (Ad5.S1-rS1Beta with an s.c. or i.n. prime-i.m. boost regimen of at a 52-week interval in 60-week-old mice) was 32-fold at week 2, which diverged to 55.7-fold and 18.4-fold at week 4 postboost, respectively, compared to those at week 0. These abundant and fast recall responses might be from memory B cells. This finding could be partially explained by previous research showing that longer intervals between SARS-CoV-2 infection and vaccination may promote a better humoral immune response in individuals previously infected with SARS-CoV-2 (36). Additionally, spike-specific memory B cells are more abundant 6 months after symptomatic onset than 1 month after onset (37), and memory B cells against SARS-CoV-2 spike actually increased between 1 month and 8 months after infection (38). It might be necessary to allow a certain amount of time for antibody somatic mutation, memory B cell clonal turnover, and the development of monoclonal antibodies that are exceptionally resistant to SARS-CoV-2 RBD mutations, including those found in the VOCs (37).

Although there were no significant differences between the groups of mice primed with Ad5.S1 s.c. and those primed by i.n. delivery, the group primed s.c. showed longer-lasting and higher GMTs after a booster injection than the group primed i.n. (Fig. 2A). These differences could be attributed to the greater systemic exposure to Ad particles upon s.c. delivery than upon i.n. delivery as well as innate immune responses potentially enhanced by the relatively invasive s.c. injections. However, it is not guaranteed that s.c. injection will be better than i.n. delivery for protection against existing and newly emerging SARS-CoV-2 variants. Various studies have reported that vaccines delivered i.n. elicited superior mucosal immunity compared to those delivered by i.m. injection and were efficient in protecting against the virus and reducing viral transmission (39–42). Moreover, a recent study of adjuvanted S1 subunit vaccines primed-boosted i.m. or primed i.m.-boosted i.n. in rhesus macaques reported that the mucosal vaccine demonstrated outstanding protection in both the upper and lower respiratory tracts by clearing the input virus more efficiently through higher dimeric IgA and IFN-α levels in bronchoalveolar lavage (BAL) fluid, although intranasal boosting elicited weaker T cell responses and lower neutralizing antibody titers (39).

In this study, high titers of serum S1 binding IgG were investigated for up to 28 weeks after booster vaccination in aged mice that were primed with Ad5.S1 1 year previously (see Fig. S1B in the supplemental material). Although the limits of IgG duration in mice may not reflect those measured in nonhuman primates or humans, this result implied that humoral immunity might be long-lasting after a booster because the IgG titers at 28 weeks postboost in G4 and G5 were approximately 6-fold and 1.7-fold higher than those at 28 weeks postprime, respectively. Indeed, boosting dramatically enhanced humoral and cell-mediated immune responses in aged mice (34). Likewise, one of the approved COVID-19 vaccines, Ad26.COV2.S, which is a single-shot-regimen vaccine protecting against severe COVID-19, induced durable immune responses that were detected up to 8 months after vaccination in humans (43). The protection provided by two doses of the mRNA BNT162b2 vaccine waned considerably after 6 months in humans. However, infection-acquired immunity boosted with vaccination remained high for more than 1 year after infection (44).

The subunit vaccine booster elicited high levels of both S1-specific IgG1 and IgG2a subclass antibodies in aged mice primed with Ad5.S1, indicating a balanced Th1/Th2 response (Fig. 2B and C). In contrast, the subunit vaccine alone induced high IgG1 with lower IgG2a levels, leading to the possibility of vaccine-associated enhanced respiratory disease (VAERD) (45). Indeed, VAERD-like pulmonary immunopathology related to Th2-biased immune responses was observed in animals vaccinated with whole-inactivated SARS-CoV vaccines (46, 47). In this study, a high level of neutralizing antibodies and a balanced Th1/Th2 immune response were induced, suggesting that a booster of a subunit vaccine after an adenoviral prime vaccine might avoid a Th2-biased immune response and the occurrence of VAERD.

Neutralization assays were frequently used as a correlate of protection following vaccination (23–26, 48). Here, we used a microneutralization test (VNT) to evaluate the function of the antibodies generated in the sera of immunized mice. The titer of neutralizing antibodies dramatically increased after a booster and neutralized other Beta and Gamma variants (Fig. 3). Our future studies will include the evaluation of the neutralization effect against the Omicron variant. Notably, a recent study demonstrated that the immune response boosted by the mRNA BNT162b2 vaccine can neutralize the Omicron variant (34). If needed, it may be possible to further improve neutralizing antibody responses with a booster of an Omicron BA.5 rS1 subunit vaccine to overcome emerging SARS-CoV-2 infections. Neutralizing antibodies against SARS-CoV-2 are effective at blocking spike-ACE2 binding to prevent infection (15, 16). As a conventional pseudoneutralization test, a competitive immunoassay for quantifying the inhibition of the spike-ACE2 interaction can be used as a surrogate for the traditional virus-based plaque reduction neutralizing assay and has reported high levels of concordance and correlation (>96%) (17, 18). In this study, we assessed animal immune responses for blocking spike-ACE2 binding using the V-Plex neutralization kit and showed that a booster in aged mice primed with Ad5.S1 could induce a significant block of the binding of ACE2 to the spike proteins of a wide range of SARS-CoV-2 variants, including Omicron variants (Fig. 5), which was correlated with the VNT_90_ (Fig. 6). In addition, our future work will include further investigations of the VNT_90_ against the spike proteins of Omicron variants.

Here, we have demonstrated the booster effect of the nonadjuvanted subunit vaccine. However, an adjuvanted subunit booster strategy is likely to have a beneficial effect on protection, particularly against distant variants such as Omicron BA.5. In fact, in nonhuman primates, the AS03-adjuvanted CoV2 preS dTM (SARS-CoV-2 prefusion spike delta transmembrane) (B.1.351) induced higher neutralizing antibody titers against the Beta variant than the nonadjuvanted vaccine in the mRNA-primed cohort (20). The AS01-like adjuvanted SARS-CoV-2 subunit vaccine enhanced the Th1 type-IgG2a isotype, neutralizing antibody, and IFN-γ-secreting T cell immune responses in both young and aged mice (49). Moreover, the combination of recombinant S protein and the CoVaccine HT adjuvant induced a balanced IgG subtype antibody response (45).

Two limitations of this study were the absence of T cell immunity testing of cellular immunity and SARS-CoV-2 challenge to assess the protection efficacy of booster vaccination. However, various studies previously reported that T cell immunity was activated after a booster (22, 39, 50, 51). Homologous and heterologous boosters in health care workers who had received a priming dose of the Ad26.COV2.S COVID-19 vaccine resulted in higher levels of T cell responses than in the nonbooster group, although the T cell responses were significantly higher with mRNA-based vaccines (91%) than with the homologous booster (72%) (22). Additionally, a booster dose of mRNA BNT162b2 elicited robust T cell responses that cross-recognized the SARS-CoV-2 Omicron variant in aged mice (34). Not only mRNA vaccines but also adenoviral vector or adjuvanted protein subunit vaccines enhanced cellular immune responses in aged mice after a boost (12, 52). Furthermore, S-specific T cell responses were positively correlated with the presence of S-specific binding antibodies (22), implying the induction of a robust T cell immune response after the rS1beta booster in this study.

As our study does not define the ability to protect against SARS-CoV-2 variants by challenge, this needs to be investigated in the future. In a Syrian hamster model of virus transmission, a prime-boost vaccine strategy using a subunit vaccine (spike HexaPro plus a cationic liposomal adjuvant) showed effective protection against SARS-CoV-2 infection (53), although the levels of antibodies for ACE2 inhibition using the MSD panel 19 ACE2 competition assay were lower than those from G4 and G5 at week 2 postboost (Fig. S1C). Notably, a recent study performed a protection experiment against a SARS-CoV-2 Omicron variant in aged BALB/c mice boosted with an mRNA vaccine (34). This natural mouse model of SARS-CoV-2 infection assessing viral replication and histopathological changes in the lung does not require genetic modifications of mice or viruses. However, this wild-mouse animal model supports infection only by SARS-CoV-2 variants that carry the N501Y mutation, including Alpha, Beta, Gamma, and Omicron (54). Therefore, it is still important to use K18-hACE2 and other hACE2-transgenic mice to investigate the pathogenicity of different SARS-CoV-2 variants (55).

Overall, our study evaluated the effect of a booster in aged mice after priming with adenoviral vaccines as a preclinical model of elderly people immunized with the currently approved COVID-19 vaccines. Our findings may have implications for further studies using the recombinant protein S1BA.5 subunit vaccine as a booster to enhance cross-neutralizing antibodies against new emerging variants of concern.

## MATERIALS AND METHODS

### Construction of recombinant-protein-expressing vectors.

The coding sequence for SARS-CoV-2 S1 amino acids (aa) 1 to 661 (56) was mutated at del144;K417N;E484K;N501Y;A570D;D614G, and a C-terminal tag known as “C-tag,” composed of the four amino acids glutamic acid, proline, glutamic acid, and alanine (E-P-E-A), was added. The sequence was also codon optimized using the UpGene algorithm for optimal expression in mammalian cells (57) and synthesized by GenScript. The construct also included a Kozak sequence (GCCACC) at the 5′ end. Plasmid pAd/SARS-CoV-2-S1Beta was created by subcloning the codon-optimized SARS-CoV-2 S1Beta inserts into the shuttle vector pAdlox (GenBank accession number U62024) at the SalI/NotI sites. The plasmid constructs were confirmed by DNA sequencing.

### Transient production in Expi293 cells.

pAd/SARS-CoV-2-S1Beta was amplified and purified using a ZymoPURE II plasmid maxiprep kit (Zymo Research). For the transfection of Expi293 cell, we used an ExpiFectamine 293 transfection kit (Thermo Fisher) according to the manufacturer’s instructions. Cells were seeded at 3.0 × 10^6^ cells/mL 1 day before transfection and grown to ~4.5 × 10^6^ to 5.5 × 10^6^ cells/mL. Mixtures of 1 μg of DNA and ExpiFectamine per mL of culture were combined and incubated for 15 min before addition to the culture at a density of 3.0 × 10^6^ cells/mL of culture. At 18 to 22 h posttransfection, an enhancer mixture was added, and the culture was shifted to 32°C. The supernatants were harvested at 5 days posttransfection and clarified by centrifugation to remove cells, followed by filtration through 0.8-μm, 0.45-μm, and 0.22-μm filters. The supernatants were either subjected to further analysis by sodium dodecyl sulfate-polyacrylamide gel electrophoresis (SDS-PAGE) and Western blotting or stored at 4°C before purification, as previously described (30, 35, 56).

### Purification of recombinant proteins.

The recombinant proteins, named rS1Beta, were purified using a CaptureSelect C-tagXL affinity matrix prepacked column (Thermo Fisher), according to the manufacturer’s guidelines. Briefly, the C-tagXL column was conditioned with 10 column volumes (CV) of equilibration/wash buffer (20 mM Tris [pH 7.4]) before sample application. The supernatant was adjusted to 20 mM Tris with 200 mM Tris (pH 7.4) before being loaded onto a 5-mL prepacked column according to the manufacturer’s instructions at a rate of 5 mL/min. The column was then washed by alternating with 10 CV of equilibration/wash buffer, 10 CV of strong wash buffer (20 mM Tris, 1 M NaCl, 0.05% Tween 20 [pH 7.4]), and 5 CV of equilibration/wash buffer. The recombinant proteins were eluted from the column by using elution buffer (20 mM Tris, 2 M MgCl_2_ [pH 7.4]). The eluted solution was concentrated and desalted with preservative buffer (PBS) in an Amicon Ultra centrifugal filter device with a 50,000-molecular-weight cutoff (Millipore). The concentration of the purified recombinant proteins was determined using a bicinchoninic acid (BCA) protein assay kit (Thermo Scientific) with bovine serum albumin (BSA) as a protein standard, and the proteins were separated by reducing SDS-PAGE and visualized by silver staining.

### Animals and immunization.

At week 52 (60 weeks old) postprime, female BALB/c mice (*n* = 5 animals per group) primed with an adenovirus-based COVID-19 vaccine (Ad5.S1) at 8 weeks of age (30) were boosted intramuscularly (i.m.) with 15 μg of rS1Beta in the thigh or PBS as a negative control. Mice were bled from the retro-orbital vein at weeks 0, 2, 4, 8, 10, 16, and 28 after booster immunization, and the obtained serum samples were diluted and used to evaluate S1-specific antibodies by an enzyme-linked immunosorbent assay (ELISA). Serum samples obtained on weeks 0, 2, and 4 postboost were also used for a VNT assay. Since aged mice develop spontaneous leukemias and other tumors, dedicated veterinarians oversaw the animals’ physical and psychological health and ruled out mice having diseases that may influence the immune responses. Indeed, one mouse in G4 at week 80 was ruled out at week 28 postboost (88 weeks old) because it was euthanized due to a tumor. Mice were maintained under specific-pathogen-free conditions at the University of Pittsburgh, and all experiments were conducted according to animal use guidelines and protocols approved by the University of Pittsburgh’s Institutional Animal Care and Use Committee (IACUC).

### ELISA.

To evaluate the expression of the SARS-CoV-2 S1Beta recombinant protein, ELISA plates were coated with chimeric MAb 40150-D003 (1:750; Sino Biological) overnight at 4°C in carbonate coating buffer (pH 9.5) and then blocked with PBS containing 0.05% Tween 20 (PBST) and 2% BSA for 1 h. The supernatants of Expi293 cells transfected with pAd/SARS-CoV-2-S1Beta were diluted 1:40 in PBST with 1% BSA and, along with standard control protein 40591-V08H (rS1H; Sino Biological) or purified rSARS-CoV-2 S1Beta, were incubated overnight at 4°C. After the plates were washed, horseradish peroxidase (HRP)-conjugated chimeric secondary MAb 40150-D001 (1:10,000; Sino Biological) was added to each well, and the plates were incubated for 1 h. The plates were then washed three times and developed with 3,3′,5,5′-tetramethylbenzidine, the reaction was stopped with 1 M H_2_SO_4_, and the absorbance at 450 nm was determined using an ELISA reader (SPECTRAmax; Molecular Devices).

To investigate the immunogenicity of SARS-CoV-2 S1Beta recombinant protein, IgG, IgG1, and IgG2a endpoint titers were measured using a laboratory-developed ELISA, as previously described (30, 35, 56).

### SARS-CoV-2 microneutralization assay.

Neutralizing antibody titers against SARS-CoV-2 were defined according to the following protocol (58, 59). Briefly, 50 μL of a sample from each mouse, starting from 1:10 at a 2-fold dilution, was added to two wells of a flat-bottom tissue culture microtiter plate (Costar) and mixed with an equal volume of 100 50% tissue culture infective doses (TCID_50_) of SARS-CoV-2 Wuhan, Beta, or Delta strains isolated from symptomatic patients, which were previously titrated. After 1 h of incubation at 33°C with 5% CO_2_, 3 × 10^4^ Vero E6 cells were added to each well. After 72 h of incubation at 33°C with 5% CO_2_, the wells were stained with Gram’s crystal violet solution for 30 min. After washing, the wells were scored to evaluate the degree of cytopathic effect (CPE) compared to the virus control. The neutralizing titer was the maximum dilution with a 90% reduction of CPE. A positive titer was ≥1:10. A negative titer was <10, which was shown as a 5 on the Y-axis of the VNT90 endpoint titer. Sera from mice before vaccine administration were always included in the VNT assays as a negative control.

### ACE2-blocking assay.

Antibodies blocking the binding of SARS-CoV-2 spike variants (Alpha [B.1.1.7], Beta [B.1.351], Gamma [P.1], Delta [B.1.617.2], Zeta [P.2], Kappa [B.1.617.1], New York [B.1.516.1], and India [B.1.617 and B.1.617.3]) to ACE2 were detected with a V-Plex SARS-CoV-2 panel 18 (ACE2) kit (Meso Scale Discovery [MSD]) according to the manufacturer’s instructions. For the inhibition of ACE2 binding to Omicron variants, we used MSD V-Plex SARS-CoV-2 ACE2 kit panel 25, including Wuhan, Omicron (BA.1), Omicron subvariants (BA.2, BA.3, BA.1+R346K, and BA.1+L452R), the Delta lineage (AY.4), Alpha (B.1.1.7), Beta (B.1.351), and France (B.1.640.2). The assay plate was blocked for 30 min and washed. Serum samples were diluted (1:25, 1:100, or 1:400), and 25 μL was transferred to each well. The plate was then incubated at room temperature for 60 min with shaking at 700 rpm, followed by the addition of Sulfo-Tag-conjugated ACE2, and incubation was continued with shaking for 60 min. The plate was washed, 150 μL MSD Gold read buffer B was added to each well, and the plate was read using the QuickPlex SQ 120 imager. Electrochemiluminescence (ECL) values were generated for each sample. Results were calculated as the percent inhibition compared to the negative control for the ACE2 inhibition assay, and the percent inhibition is calculated as follows: % neutralization = 100 × [1 − (sample signal/negative-control signal]).

### Statistical analysis.

Statistical analyses were performed using GraphPad Prism v9 (GraphPad Software, San Diego, CA). Antibody endpoint titers and neutralization data were analyzed by the Kruskal-Wallis test followed by Dunn’s multiple-comparison test. Significant differences are indicated by asterisks in the figures (*, *P* < 0.05). Comparisons with nonsignificant differences are not indicated. Correlations between the V-Plex ACE2-blocking and VNT_90_ or IgG endpoint titers and VNT_90_ values were determined using correlation analysis and calculations of Spearman coefficients and 95% confidence intervals (CIs).
