# Supplementary material for: SARS-CoV-2 S1 Subunit Booster Vaccination Elicits Robust Humoral Immune Responses in Aged Mice
Source: Microbiol Spectr. 2023 May 10;11(3):e04363-22. doi: 10.1128/spectrum.04363-22 (PMC10269910; doi:10.1128/spectrum.04363-22)
Supplement: Supplemental file 1 — Fig. S1 to S3. Download spectrum.04363-22-s0001.pdf, PDF file, 0.8 MB [file spectrum.04363-22-s0001.pdf]

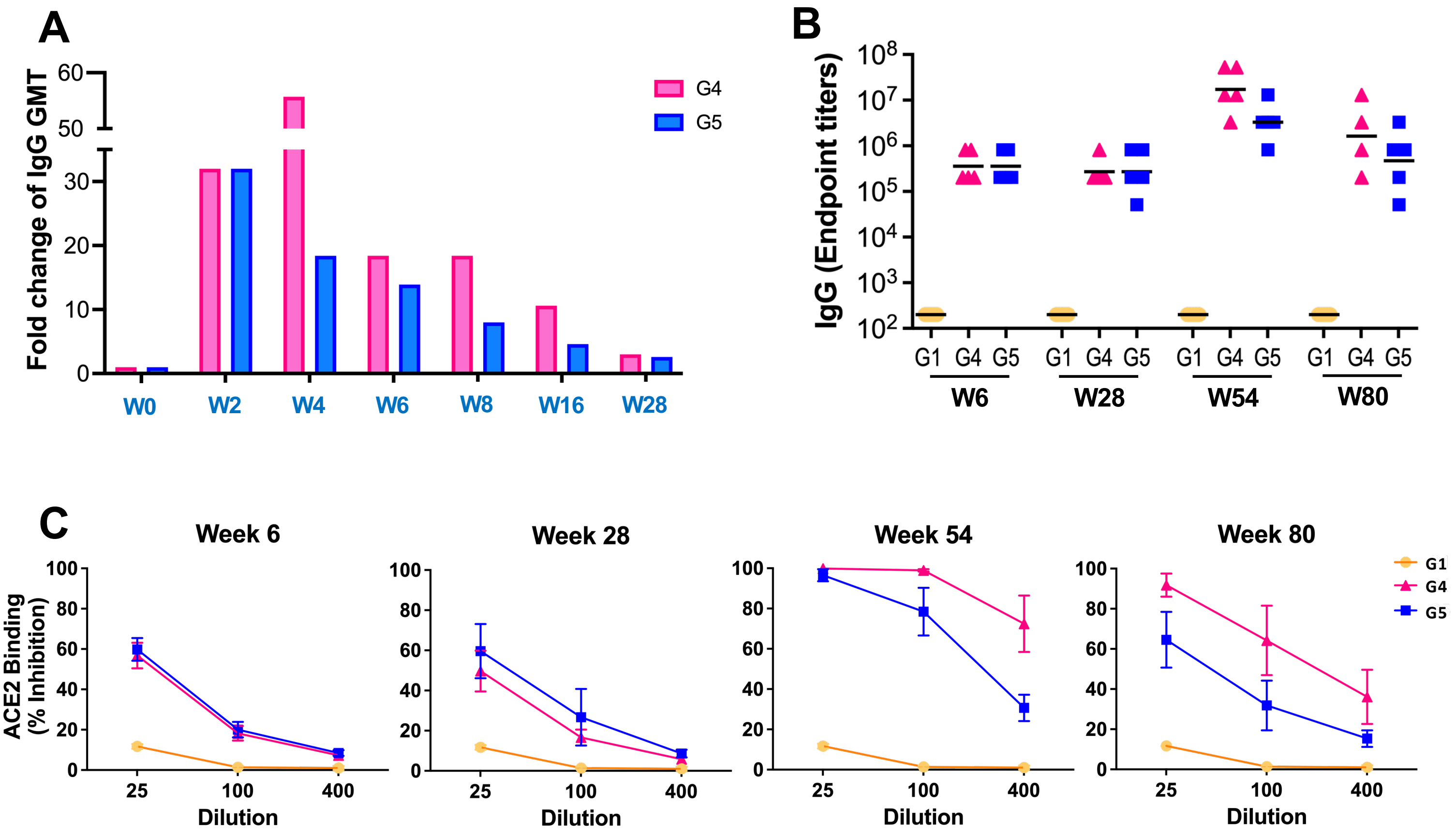

FIG S1 Comparison of serological responses to S1 from mice of G1, G4, and G5 post-prime and post-boost. Mice were primed with adenoviral vaccine subcutaneously or intranasally and boosted with SARS-CoV-2-S1Beta recombinant proteins at a one-year interval, and reciprocal serum endpoint dilutions of S1- specific IgG were measured by ELISA. (A) Fold change of reciprocal serum endpoint dilutions of S1- specific IgG in G4 (pink box) and G5 (blue box) at weeks 0, 2, 4, 6, 8, 16, and 28 post-boost compared to those at pre-boost. (B) Serum endpoint titers of S1- specific IgG were assessed at highest time point (weeks 6 and 54) and at week 28 post-prime or post-boost (N=5 per group, except at week 80 G4 N=4). Horizontal lines represent geometric mean antibody titers (GMT). Significance was determined by Kruskal-Wallis test, followed by Dunn's multiple comparisons ($*p < 0.05$). (C) ACE2 binding inhibition (%) at weeks 6, 28, 54, and 80 against Wuhan in dilution 1:25, 1:100, and 1:400. Data show means ± standard error of the means (SEM) of G1 (peach circle), G4 (pink triangle), and G5 (blue square).

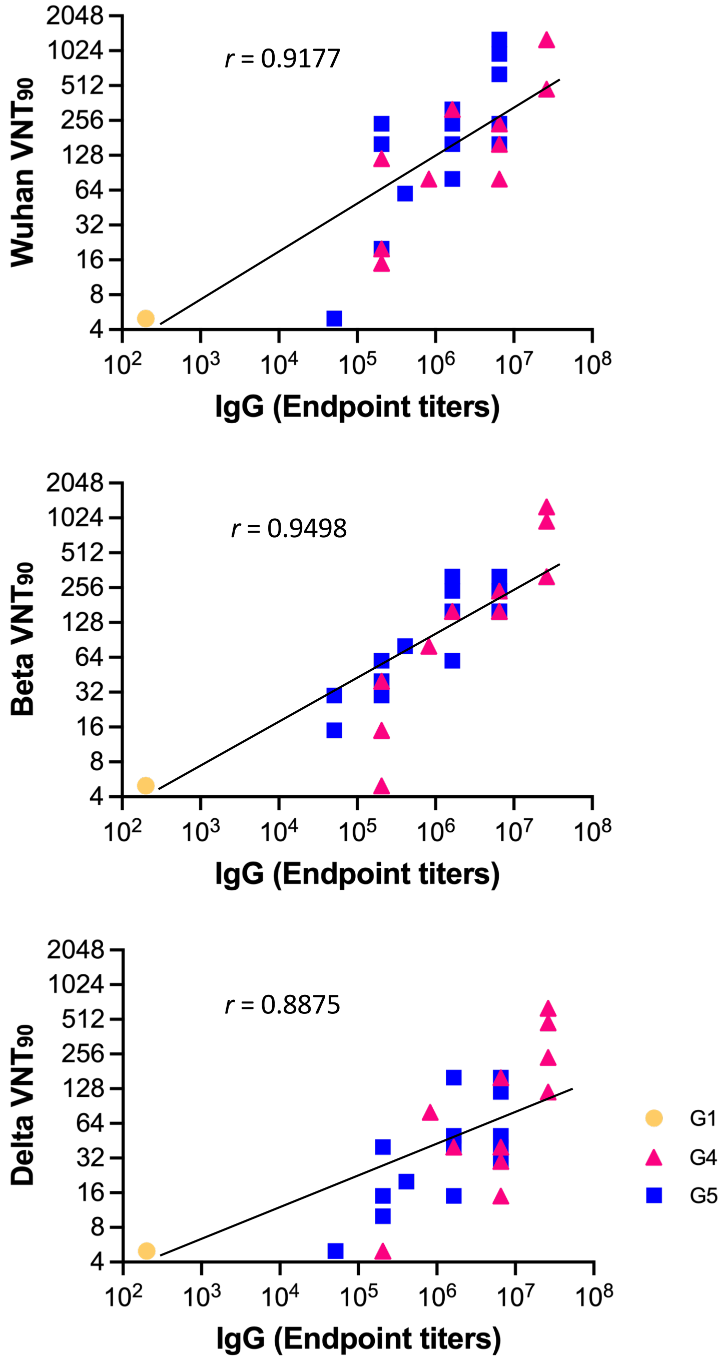

FIG S2 Correlation between the VNT_{90} and SARS-CoV-2-S1-specific IgG titer. The correlation between VNT_{90} (Log_2) against Wuhan, Beta (B.1.351), and Delta (B.1.617.2), and S1-binding IgG endpoint titers (Log_{10}) in all animals from G1 (peach circle), G4 (pink triangle), and G5 (blue square) at week 0, 2, and 4 post-boost is shown. The lines represent the regression line of all samples. Each symbol represents an individual mouse. Correlation analysis and calculation of Spearman's correlation coefficients were performed using GraphPad Prism v9.

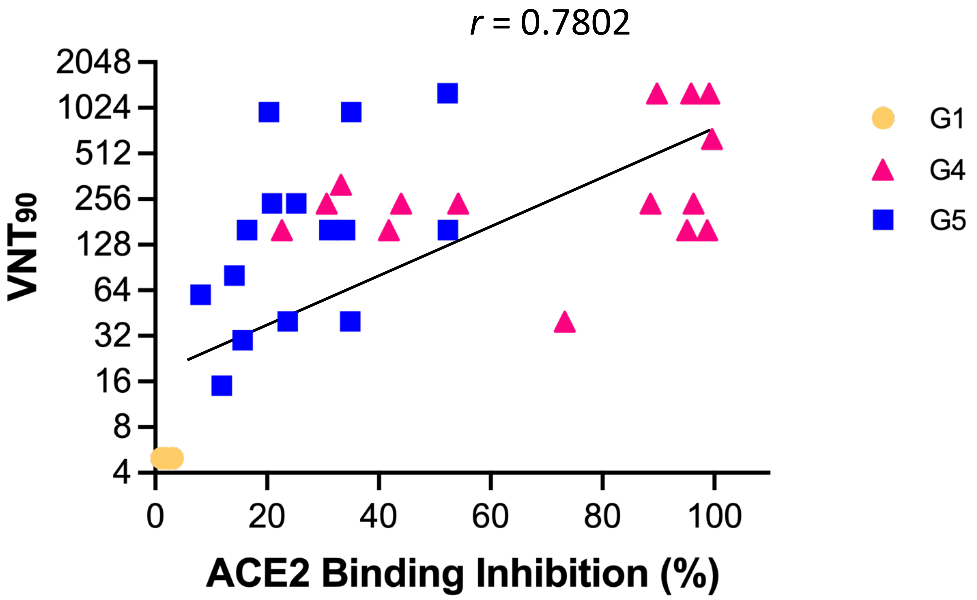

FIG S3 Correlation between VNT_{90} (Log_2) against Wuhan, Beta (B.1.351), and Delta (B.1.617.2) and ACE2 binding inhibition (%) of 1:400 diluted sera from all animals of G1 (peach circle), G4 (pink triangle), and G5 (blue square) at week 2 post-boost is shown. The line represents the regression line of all samples. Correlation analysis and calculation of Spearman's correlation coefficients were performed using GraphPad Prism 9.
